# Special Issue: “Advances in Structural Mechanics Modeled with FEM”

**DOI:** 10.3390/ma14040780

**Published:** 2021-02-07

**Authors:** Angelo Marcello Tarantino, Carmelo Majorana, Raimondo Luciano, Michele Bacciocchi

**Affiliations:** 1 Department of Engineering “Enzo Ferrari” (DIEF), University of Modena and Reggio Emilia, Via Vivarelli, 41125 Modena, Italy; 2 Department of Civil, Environmental and Architectural Engineering (DICEA), University of Padova, Via F. Marzolo, 35131 Padova, Italy; 3 Engineering Department, University of Napoli Parthenope, Via Ammiraglio Ferdinando Acton, 80133 Napoli, Italy; 4 Dipartimento di Economia, Scienze e Diritto (DESD), University of San Marino, Via Consiglio dei Sessanta, 47891 Dogana, San Marino

The current Special Issue entitled “Advances in Structural Mechanics Modeled with FEM” aims to collect several numerical investigations and analyses focused on the use of the Finite Element Method (FEM). The undeniable spread of this methodology in the recent decades is due to the recent improvements of high computational resources and the large-scale deployment of increasingly powerful computers, which have allowed to develop numerical approaches related to various applications. This situation has represented the key aspect that led to the birth of this Special Issue.

It is well known that many structural and physical problems are not suitable to be solved analytically. Therefore, appropriate numerical methods have to be developed to get approximate but accurate solutions. Precisely, one of the most typical approaches that can be employed to this aim is the Finite Element Method, which is characterized by very good accuracy and reliability. In addition, it can be easily implemented to deal with several applications in many engineering fields. In fact, it is easy to find numerical analyses solved by means of the FEM in civil, mechanical, and aerospace engineering.

The Guest Editors would like to emphasize their enormous gratitude to the Editors-in-Chief of Materials for the fantastic opportunity to manage this Special Issue. Likewise, all the authors coming from many different countries who contributed to the success of the present Special Issue are gratefully acknowledged for their papers characterized by very high quality. The reviewers should be also mentioned for their support, providing acute observations which undoubtedly improved the submitted manuscripts. Finally, the Section Managing Editor, Ms. Floria Liu, is acknowledged for accurately and constantly managing the editorial process.

The success of this venture is proved by the twelve papers collected and published in just over a year and a half. A brief review of these papers is presented below to highlight the multidisciplinary and the quality of these researches.

A two-node beam element was developed to analyze the static bending of functionally graded (FG) beams by Nguyen et al. [1]. The quasi-3D beam theory which the formulation is based on allowed to avoid the shear-looking issue without selective or reduced integration. A power-law expression was introduced to characterize the graded mechanical properties of the structures. Many parametric tests were illustrated to emphasize the effect of the graded features on the bending response.

A finite element model was developed by Zhang et al. [2] to investigate the gas flow properties in the coal seam. The starting point of their research was the fact that coal is characterized by a large number of fractures, whose spatial distribution patterns may follow some macroscopic statistical laws. Therefore, they described the coal seam as a two-dimensional stochastic fracture network introducing various fracture geometric parameters to represent a fracture. The paper proved that the permeability of the coal seam is emphasized with the increase of fracture density, length, aperture.

The natural frequencies of sandwich plates were analyzed in the paper by Bacciocchi et al. [3] by using a finite element formulation based on the Reissner-Mindlin theory including the zig-zag effect. The plates were made of a damaged isotropic soft-core and two external orthotropic skins reinforced by randomly oriented Carbon nanotubes. A three-phase method combining the Eshelby–Mori–Tanaka scheme and the Halpin–Tsai approach was developed to evaluate the overall mechanical features of the composites. Various parametric investigations were carried out to highlight several effects on the dynamic response.

The fracture process of soda-lime glass under impact loading was investigated numerically in the paper by Li and Wei [4], establishing a rate-dependent cohesive zone model implemented in a commercial finite element code. The accuracy of the fracture model was enhanced by taking into account the strain rate effect and the tensile-shear mixed mode fracture. The paper proved that the rate-dependent cohesive zone model was more able to describe the impact failure features of a monolithic glass plate if compared to a cohesive zone model without the inclusion of the strain rate. The effects of strain rate sensitivity coefficient, mesh size and impact velocity were also discussed in the research.

Gilewski and Pełczyński developed a four-noded finite element for the analysis of thick plates made of (FG) materials [5]. A coordinate change based on NURBS functions was used to describe arbitrarily shaped domains. Full coupling of the membrane and bending states was included in the formulation, which was embedded in a commercial finite element code. A good convergence characterized their finite element in dealing with the analysis of nonhomogeneous FG plates.

The finite bending of prismatic hyperelastic solids was investigated in the paper by Falope et al. [6] taking into account the anticlastic effect. The corresponding finite element formulation was developed and the numerical results were compared with the ones obtained analytically. The theoretical framework was fully nonlinear, assuming finite displacements and deformations. Analogously, the constitutive law was based on the Mooney–Rivlin nonlinear model for rubberlike materials. The analyses aimed at discussing the Eulerian slenderness and the compactness index of the beam cross section, which were involved in the definition of the Searle parameter for bent solids.

Gnoli et al. [7] discussed the validity of commercial finite element codes that implement the classical isotropic beam model for studying some equivalent composite configurations. A simple approach to deal with composite beams as isotropic configurations was developed by removing those complexities related to the modelling of composite materials when 2D and 3D finite element approaches were used. To this aim, the stiffness matrix of the equivalent isotropic beam was computed by means of an analytical approach valid for various laminated configurations used in typical engineering applications.

Dunaj et al. [8] focused their research on the development of a methodology able to model the natural frequencies, mode shapes, and receptance functions of machine tool steel welded frames filled with strongly heterogeneous polymer concrete, by employing low-dimensional models based on the rigid finite elements method (RigFEM). The numerical solutions were validated with respect to the experimental results, highlighting the accuracy of the proposed approach in terms of relative error.

Masonry structures in the plane stress state were the main topic of the paper by Bilko and Małyszko [9]. In particular, their work dealt with orthotropic masonry at the material level and presented the implementation of a continuum structural model for the analysis in a finite element code. The mathematical elastoplasticity theory represented the theoretical framework for developing the constitutive relations, assuming small displacements and deformations. The authors proposed a generalization of the Hoffman orthotropic failure criterion in the plane stress state.

The study by Ito et al. [10] aimed at evaluating the internal stresses in marble slabs experiencing bowing phenomenon due to thermal loading, taking into account the true deformed shape in continuum media and defining the stress–strain relationship in the hypothesis of finite displacements. The results obtained by means of a finite element based procedure proved that that transient heat flux should induce higher stresses than just applying greater gradients of temperature in steady flux, which could clarify the larger decohesion through width occurring in bowing tests. They emphasized that previous published results concerning the mechanical behavior of bowing based on classical beam theories could turn out to be inadequate when the internal stresses were computed.

The paper by Rothe et al. [11] highlighted the enormous potentialities of additive manufacturing, especially as far as the advanced acoustic design measures like Acoustic Black Holes are concerned. They specified that the continuous alignment of the mechanical impedance could be accomplished by means of the layer-wise material deposition by setting different filling patterns and levels of filling which characterized the technique. To this aim, experimental tests were carried out considering several configurations in order to identify the material parameters in dependency on the frequency and the thickness. A 3D finite element model was developed for the parameter fitting, proving the reliability of their results.

Finally, Mathern and Yang presented a modeling strategy and many practical recommendations for the finite element analysis in nonlinear regime of reinforced concrete (RC) structures based on parametric studies of critical modeling choices [12]. The aim of the paper was the reliable prediction of flexural responses of RC elements, focusing on cracking behavior and crushing failure. These aspects should be taken into account when more complex cases are modeled, such as RC beams bonded with fiber reinforced polymer laminates. Their results were validated by means of experimental tests, as well.

## Data Availability

Data sharing not applicable.
